# Scaling up the “24/7 BHU” strategy to provide round-the-clock maternity care in Punjab, Pakistan: a theory-driven, coproduced implementation study

**DOI:** 10.1186/s12961-022-00944-w

**Published:** 2022-12-28

**Authors:** Sarah Salway, Zubia Mumtaz, Afshan Bhatti, Amy Barnes, Jeremy Dawson, Gian Singh Jhangri

**Affiliations:** 1 Faculty of Social Sciences, University of Sheffield, Elmfield Building, Northumberland Road, Sheffield, S10 2TU UK; 2 School of Public Health, University of Alberta, 3-300 Edmonton Clinic Health Academy, 11405-87 Ave, Edmonton, AB T6G 1C9 Canada; 3 Real Medicine Foundation, 328 Service Road, E-11/4, Islamabad, Pakistan; 4 Management School, University of Sheffield, Conduit Rd, Sheffield, S10 1FL UK

**Keywords:** Scale-upprogrammes, Maternal healthcare service, Rural, Low- and middle-income countries, Coproduction of knowledge

## Abstract

**Background:**

Pakistan’s maternal mortality rate remains persistently high at 186/100,000 live births. The country’s government-run first-level healthcare facilities, the basic health units (BHUs), are an important source of maternity care for rural women. However,BHUsonly operate on working days from 8:00 am to 2:00 pm. Recognizing that this severely constrains access to maternity services, the government is implementing the “24/7 BHU” initiative to upgrade BHUs to provide round-the-clock care. Although based on a successful pilot project, initial reports reveal challenges in scaling up the initiative. This implementation research project aims to address a key concern of the Government of Punjab: How can the 24/7 BHU initiative be successfully implemented at scale to provide high-quality, round-the-clock skilled maternity care in rural Punjab?

**Methods:**

The project consists of two overlapping work packages (WP). WP1 includes three modules generating data at the directorate, district and BHU levels. Module 1 uses document analysis and policy-maker interviews to explicateprogrammetheory and begin to build a system model. Module 2 compares government-collected data with data generated from a survey of 1500 births to assess BHU performance. Module 3 uses institutional ethnographies in 4–5 BHUs in three districts to provide a detailed system for understanding and identifying processes that influence scale-up. WP2 includes two modules. First, two workshops and regular meetings with stakeholders integrate WP1 findings, identify feasible changes and establish priorities. Next, “change ideas” are selected for testing in one district and 2–3 BHUs through carefully documented pilots using the PDSA (plan–do–study–act) improvement approach. An integrated knowledge translation approach will engage key policy and practice stakeholders throughout the project.

**Discussion:**

This theory-driven implementation research project willcoproducesignificant new understandings of the wider system in which the 24/7 BHU initiative is being implemented, and actionable knowledge that will highlight ways the implementation processes might be modified to enable BHUs to meet service provision goals. This study will also produce insights that will be relevant for other South Asian and low- and middle-income countries (LMICs) that experience similar challenges of programme scale-up and delivery of maternal health services to remote and marginalized communities.

## Background

This project addresses a key concern of the Government of Punjab Health Department: How can the 24/7 Basic Health Unit (BHU) initiative be successfully implemented at scale to provide high-quality, round-the-clock skilled maternity care in its first-level care facilities in remote, rural Punjab?

With a maternal mortality ratio (MMR) of 186/100,000 live births, Pakistan is one of six countries that account for 50% of all maternal deaths globally [1, 2]. Pakistan failed to meet its Millennium Development Goal (MDG) 5 target [3], and large urban–rural inequalities persist in access to skilled maternity care and MMR [4]. Continuous skilled birth attendance backed by emergency obstetric and newborn care (EmONC) is an acknowledged pillar of safe childbirth [5]. While Pakistan has extensive healthcare infrastructure, structural system deficiencies hinder delivery of quality services, particularly in the public sector serving poor women in rural areas [6]. A key limitation is that BHUs, the first-level healthcare facilities designed to provide basic EmONC services, are only open from 8:00 am to 2:00 pm [7].

To address these concerns, the Chief Minister’s Health Initiative for Attainment & Realization of Millennium Development Goals (CHARM) tested a donor-supported intervention aimed at upgrading BHUs to provide round-the-clock basic EmONC in seven districts in Punjab [8]. Evaluation of the pilot showed some important successes. NonfunctionalBHUswere revived, and round-the-clock availability of midwives improved. A significant increase in rates of skilled birth attendance was reported (27% in pilots vs 14% in control BHUs) [9]. Some problems remained, however, including drug stock-outs [8].

Based on this success, the Government of Punjab scaled up the CHARM pilot, incorporating it into its Health Sector Reform Strategy (2012–2018). This scale-up, known as the 24/7 BHU initiative, is being implemented through the Integrated Reproductive Maternal Newborn Child Health & NutritionProgramme(IRMNCHN). It aims to upgrade at least one third of BHUs in all 36 districts to provide round-the-clock basic EmONC services and to ensure that district headquarter hospitals provide high-quality, comprehensive EmONC [9]. To date, 709 BHUs have been upgraded. However, implementation status monitoring reports and conversations with government officials suggest significant challenges and a need to better understand obstacles and enablers of successful implementation across local settings [10].

Challenges during scale-up are not unusual. A growing body of literature documents the complexity of integrating pilot interventions into wider health systems of low- and middle-income countries (LMICs) at scale [11, 12]. Numerous examples are found of promising initiatives that fail when scaled up, often because of a failure to adequately understand and respond to contextual variation in health systems and insufficient “ownership” by national and local stakeholders [13]. Hawe et al. argue that a new initiative is not an isolated event, but a disruption to a pre-existing complex ecosystem made up of multilayered components that are interrelated in nonlinear ways [14]. These complex systems are emergent and unpredictable, so that the intended effects of an intervention cannot be taken for granted. Both favourable and unfavourable system changes may result from introduction of the new initiative [15, 16]. However, complex systems are also adaptive, with some actors having greater agency by virtue of power and resource distributions. As such, “deep structures and processes” of society must be recognized and interrogated, since these tend to produce predictable “winners” and “losers” even though the exact manifestations vary [17].

The challenge of scaling up complex healthcare interventions is increasingly recognized as a key area of health systems enquiry [18]. This theory-driven implementation research project aims to generate a detailed understanding of the scale-up of the 24/7 BHU initiative as it is unfolding on the ground across a variety of local settings. The research will also support the success of the initiative by coproducing with policy and practice stakeholders promising avenues for refinement of theprogrammegoing forward.

## Research questions

1. What is theprogrammetheory underlying the 24/7 BHU initiative? What assumptions and understandings of the wider system are embedded in it?
2. To what extent are key performance indicators of the 24/7 BHU initiative being met? How does performance vary across settings and between subgroups of women?
3. How does the 24/7 BHU initiative work in the dynamics of different settings? What system processes are supporting or undermining successful scale-up?
4. How does the 24/7 BHUprogrammetheory relate to systems’ influences on scale-up? What are key areas of misalignment?
5. How can system elements and processes bemanoeuvred to improve implementation of the initiative overall and for subgroups of poorly served women?
6. What key system elements and processes may need to be monitored and addressed to sustain the initiative?

## Methods

### Theoretical framework

Our theoretical framework integratessocio-ecologicalsystems thinking with a practical implementation science framework and tools from health improvement science. Exploring the scale-up of the 24/7 BHU initiative from a systems perspective implies looking carefully at what happens when the initiative is rolled out across the province. It means exploring the interplay of the system elements with the intervention components and the processes that ensue—particularly those that amplify or dampen intended mechanisms—and the potentially varied trajectories and outcomes across local settings. Such a framework draws attention to proximal, intermediate and distal processes and to both the “hardware” and the “software” of the health system. So, while mapping the core health system “building blocks” [19] is essential, so too is understanding the shifts that ensue in the power, roles, relationships, understandings and motivations of key actors within the system as the scale-up proceeds. Furthermore, sociocultural values and norms are key system elements to consider [20, 21]. Health services are social institutions, culturally embedded and politically contingent, replicating the norms and ideologies of wider society [22]. Importantly, much prior work in Pakistan underscores the impact of the gender order on the health ecosystem, shaping the understandings and behaviours of managers, practitioners, patients and family members [23, 24]. Similarly, socioeconomic and caste (zaat) hierarchies are relevant, yet rarely addressed, in health services research and policy development in Pakistan. A systems approach also recognizes that interventions and initiatives are not fixed over time and place, but rather that change occurs both because of deliberate policy direction and via interpretation and “mutation” at the local level.

Systems thinking is challenging, and the various elements and processes within the health ecosystem that are relevant to understanding the scale-up of the 24/7BHU initiative are potentially overwhelming. We have therefore selected an implementation tool—the revised version of Promoting Action on Research Implementation in Health Services (PARiHS)—to support the engagement of government stakeholders in the analytical process. PARiHS can be a useful tool, providing a structure within which to make sense of the complexity of implementation, determine critical factors and processes affecting success, and identify actionable strategies for improvement [25]. PARiHS identifies three interacting elements that shape implementation. Evidence [E] refers to “codified and non-codified sources of knowledge” that key stakeholders have, context [C] refers to characteristics of the environment in which the initiative is implemented, and facilitation [F] refers to the ways in which deliberate support may promote implementation (e.g. helping people to shift attitudes, skills and ways of working). A helpful operational guide to PARiHS has been produced which sets out a comprehensive framework of prompting questions through which to interrogate the unfolding nature of an initiative [25]. The tool therefore provides a useful starting point and has been found to be intuitively appealing to policy-makers [25], who may naturally view any new initiative as separate from the context into which it is introduced and focus their attention on the resources and actions they view as within their own remit. We will use the PARiHS framework to consider the complex, dynamic and iterative interactions between the three elements (E, C and F). We will begin to map their interrelations and identify the ways in which they shape, and are shaped by, the wider system. In line with system perspectives, this mapping process will involve a temporal element, exploring the scale-up “journey” over time. In so doing, we will be mindful of the possibility that the 24/7 BHU initiative may change the boundaries of the health system (e.g. by setting up relationships with new community actors) and may produce unanticipated effects (such as the displacement of activities). We will also consider that factors that support or undermine rollout in the early days (e.g. fit between the initiative and provider role understandings) may differ from those that are sustaining the initiative over time. Similarly, objectives and outcomes of the initiative may need to be revised prospectively [25].

In the later stages of the project, we will combine our systems thinking and the PARiHS framework with tools from health improvement science—PDSA (plan–do–study–act) cycles—to plan, pilot and evaluate modifications to the 24/7 BHU initiative. PDSA is a pragmatic, structured learning approach to testing changes in complex systems that recognizes the influence of local settings and unpredictability of outcomes [26, 27]. This approach puts policy-makers and practitioners at the centre of the improvement process [28].

### Detailed project description

**Fig. 1:**
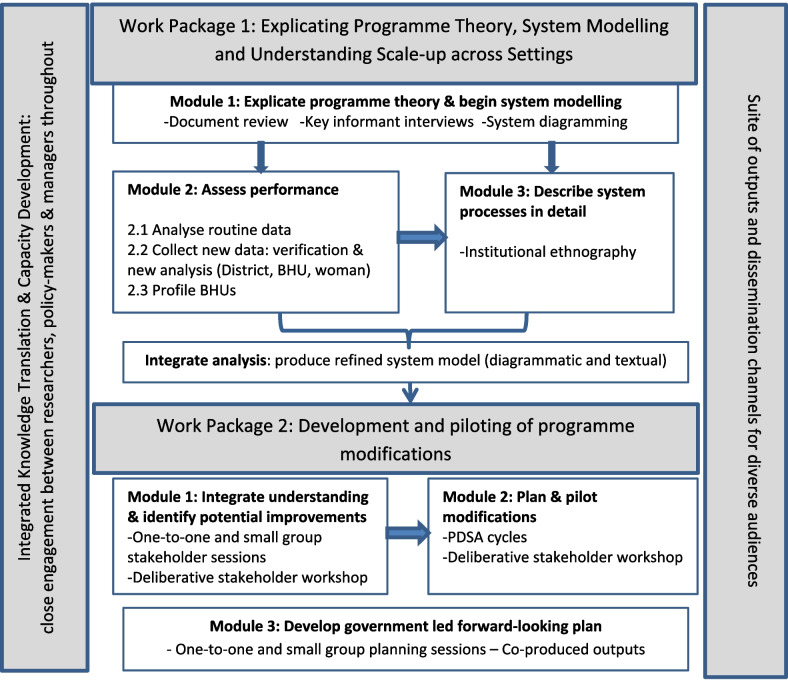
Project design

The project consists of two overlapping work packages (WP). WP1 will explicate programme theory, assess performance, describe how the initiative plays out in different settings and identify system processes influencing scale-up. WP2 will take this integrated knowledge to develop and pilot improvement approaches. An integrated knowledge translation approach [29] will engage key policy and practice stakeholders throughout the project (Fig. 1).

### WP1

A mix of quantitative and qualitative research methods will explicate the theory of the 24/7 BHU initiative, examine its operationalization across varied local settings and develop a system model of the processes that are shaping scale-up. WP1 will consist of three modules. Data will be generated at the BHU, district and directorate levels. Directorate-level data will be collected from the Provincial Department of Health in Lahore. District- and BHU-level data generation will focus on those 19 Punjab districts that have implemented the 24/7 initiative for two years or more and within which at least 20 BHUs have been upgraded (i.e. those where it can be considered that the initiative is underway).

### WP1—module 1: programme theory explication and initial system modelling

Addresses research question:

*Q1: What is the programme theory underlying the 24/7 BHU initiative? What assumptions and understandings of the wider system are embedded in the theory?*

We draw on Rossi’s definition of programme theory as a “construction of a plausible and sensible model of how a program is supposed to work” and one which “provides causal links between the operation of the program and its intended effects” (p. 134) [30]. This module will explicate, in all its complexity, the elements and processes of the 24/7 BHU initiative. It will articulate the design of the scale-up, the processes, inputs, outputs, outcomes and costs of the initiatives. In addition, it will begin to map out the wider socio-ecological system—to situate the deliberate activity of the initiative within a wider set of elements and processes. We will start to consider the complex dynamics of the initiative as it is rolled out, for example, being open to the possibility of simultaneous pathways to impact or alternative causal strands as the initiative plays out in different local settings. We will also look for evidence of reinforcing and dampening feedback loops [31].

Data will be generated through a document review and interviews, both informed by the conceptual framework described above. Documents to be reviewed include grey literature related to the 24/7 BHU initiative; Punjab Health Department policy and planning documents; Policy and Strategic Planning Unit (PSPU) monitoring reports; and minutes of provincial and district-level meetings. As aspirational documents, policy and planning documents are a key source of “deliberate and conscious statements of policies and strategies at particular points in time and can at the very least be regarded as public avowals of commitment to certain objectives and even values” (p. 261) [32]. A semi-structured template will be designed and employed systematically to extract content across the documents.

To further refine the programme theory, a series of theory-driven in-depth key informant interviews, individually and in groups of 2–4, will be conducted with 20–25 provincial and district-level policy-makers and programme managers. Respondents may include the Director-General Health, directors of the 24/7 BHU implementation unit, the IRMNCHN programme and the PSPU. Key personnel working for international organizations and donors directly associated with the 24/7 BHU initiative, specifically the World Bank and the Foreign, Commonwealth and Development Office (FCDO), will also be interviewed. As the analysis proceeds, it will identify a small number of respondents who are particularly engaged in the process for follow-up interviews to refine and consolidate the analysis. Interviews will engage respondents in critical interrogation of the initiative and its performance, drawing on the PARiHS framework where helpful, and ensuring sufficient openness for respondents to refine, refute and redefine the emergent theory and system model.

*Data management and analysis* Documents and interview transcripts (recorded subject to respondent consent) will be uploaded to the computerized textual analysis package Quirkos [33]. Analysis will involve an iterative process of theory-gleaning, theory-refining and theory consolidation. First, interpretative analysis of the documentary sources will be used to develop a draft formulation of programme inputs, outputs and outcomes; causal processes linking these elements; and any contextual factors identified as potential moderators of these relationships. This analysis, supported by the PARiHS framework, prior evidence on the Pakistani maternal healthcare system and investigators’ extensive contextual experience, will inform development of the interview guide. Following completion of the interviews, analysis of transcripts will first focus on extracting segments that speak directly to elements of the draft programme theory, redrawing, confirming or refining it, in some way. Subsequently, we will map evidence on interrelationships between programme components, wider influences, feedback loops and unanticipated effects, thereby beginning to extend the theory towards an early system model. We will adopt a coding system that maps interview segments to system elements and processes and allows for an audit trail of the iterative process of building explanations and refining the model. Since these initial interviews will also include more open-ended, respondent-led discussion around broader issues of maternal health policy and service delivery in Pakistan, we will complement the analysis described above with an inductive, thematic analysis using line-by-line coding and retrieval approaches. This analysis will seek to uncover “softer” system characteristics, including latent meanings, incentives, relationships and power structures. The product of module 1 will be visual representations and textual descriptions of the early system model. We will explore various ways of engaging policy stakeholders with the emerging understanding, including casual loop diagrams, figures employing the PARiHS E, C and F categorization, vignettes and textual summaries. The analysis will highlight gaps and embedded assumptions within the initiative that require exploration on the ground. Inconsistencies, if any, between understandings of the 24/7 BHU initiative held by different stakeholders within the system will also be noted.

### WP1—module 2: assessing patterns of performance

Addresses research questions:

*Q2: To what extent are the key performance indicators of the 24/7 BHU initiative being met in Punjab? How does performance vary across settings and between subgroups of women?*

*Q3: How does the 24/7 BHU initiative work in the dynamics of different settings? What system processes are supporting or undermining successful scale-up?*

We adopt an efficient design for WP1 module 2, combining analysis of data from the Punjab Health Department’s routine monitoring system alongside the collection of new data.

### Analysis of Punjab Health Department routine data

The Punjab Health Department monitors the 24/7 BHU initiative through data routinely collected and reported by the PSPU, a monitoring unit operating at arm’s length from the implementation unit (IRMNCHN). This monitoring arrangement—as an audit-and-feedback system [34]—is an important element of the 24/7 BHU initiative that will form part of the programme theory and will be examined in detail as described below in WP2-module 3. In addition, it offers a useful means by which dimensions of performance of the 24/7 BHU initiative can be assessed. The Punjab Health Department and PSPU have granted our research team access to the monitoring data, and this will enable a series of analyses that extend the PSPU’s routine reporting and provide additional insight into the progress of the scale-up. Precise details of the analyses and the insights these can offer will be agreed via early stakeholder meetings and a workshop in which the programme theory will be discussed (see WP2 below). However, early discussions have clarified available indicators and identified some key areas for investigation. Analyses will examine (i) data completeness, consistency and plausibility, and (ii) variation in performance across BHUs and across districts.

Currently, the key performance indicator for the 24/7 BHU initiative is facility functionality around the clock. Since January 2017, birth notifications have been sent by SMS (Short Message Service) text message in real time by midwives to the local monitoring and evaluation (M&E) person, the M&E manager and the district public health officer. These data will allow for examination of the distribution of reported birth timing. Exploration of 24-hour birth patterns will provide an indication of whether deliveries are being reported as taking place at BHUs after dark (taking into consideration seasonality). Evidence indicates that natural births do not happen evenly distributed around the clock, with 38% of births naturally occurring within the nighttime hours of midnight to 8:00 am [35]. We will also examine birth volumes in comparison with expected numbers based on demographics and prior survey findings on the proportion of facility-based births. The Health Department collects further information relevant to the availability of skilled birth attendants via the “Facility Analysis Tool” administered by district supervisory officers on a monthly basis. Data on presence, phone contact and place of residence of skilled staff will be analysed.

Indicators of facility quality are also important, and will be receiving increased attention by the Health Department as the programme is scaled up. The routine system currently collects indicators of BHU functionality including stock availability (e.g. blood haemoglobin strips and metres; essential medicines); equipment (e.g. thermometers; electronic scale); documentation (e.g. referral records); and facility “outlook” and maintenance (e.g. signage; toilets; drinking water; cleanliness) [36].

Descriptive analyses will be performed at the BHU, district and province levels. We will work closely with the technical director of the PSPU (Government of Punjab) to define the analyses and guide data handling, presentation and interpretation of results. Trends over time will be examined. A particular interest will be to examine the degree of variation in key performance indicators within and between districts. Bivariate and multivariable analyses will be performed to test key hypotheses regarding factors operating at the BHU and district levels that support (or undermine) performance. For example, it might be hypothesized that better physical BHU conditions would be positively associated with regular personnel attendance. A detailed analysis plan will be informed by the programme theory and system modelling undertaken with government stakeholders in module 1.

Recognizing the newness of the programme and the challenging objective of providing round-the-clock high-quality care (with significant demand- and supply-side obstacles), accurate reporting of birth timing and other indicators of programme functioning is likely to be affected by both administrative error and some degree of fraud. We will therefore undertake verification exercises (see below). “Margin of error” statements will then be incorporated into the results based on the Punjab Health Department monitoring system data. Data will be analysed using Stata 17 software [37].

### Verification and supplementation of Punjab Health Department data via new data collection at the district and BHU levels

The nature and objectives of the 24/7 BHU initiative dictate a three-level cluster sample in which a sample of districts is first drawn from the whole province, followed by a sample of BHUs from within each district, and finally a sample of women (births) is drawn from within each BHU. The ultimate outcomes relate to women’s access to good-quality care when needed, but important determinants of these outcomes may operate at different levels within the system. In drawing our sample, there is a trade-off to be made between the number of districts and the number of BHUs. The sample could consist of fewer districts and more BHUs per district, or more districts with fewer BHUs within each, with concomitant implications for the power to detect associations between BHU-level and district-level factors and programme performance indicators. Findings from our analyses of the Health Department routine data, and our prior systems modelling work, will therefore guide our sampling approach. In particular, working closely with Health Department stakeholders, we will identify key factors at the BHU and district levels that are believed to be (i) influential, (ii) variable and (ii) potentially modifiable. This assessment will inform the appropriate balance of districts and BHUs in the sample. Maintaining this design flexibility is important both to ensure that the emerging understanding of the system is taken on board and to promote ownership of the project and its findings among government stakeholders.

At this stage we have therefore determined the size of the woman (birth)-level sample that is desirable. Taking the proportion of births that receive “good” care (assessed via achievement of a threshold on a composite measure) as the outcome, sample size calculations indicate that a sample of 1500 women (births) is sufficient to detect a small standardized difference between subgroups of women defined by key characteristics (such as educational level) with 90% power (a series of calculations were run assuming varied splits across comparator groups, an α of 0.05 and a design effect of 1.5) [38]. Similarly, this sample size will be adequate to detect important differences in the proportion of deliveries occurring at night (as an indicator of round-the-clock access to care). We have currently budgeted for data collection for this number of births (as well as other data as described below) for a geographically dispersed sample over the north and south of the province (recognizing important variation in socioeconomic development) and assuming data collection for a minimum of 30 women (births) per BHU. This would then allow data collection from up to 50 BHUs. If the analysis of routine data suggests that BHU variation is of key importance, then we will ensure that this maximum number of BHUs is included, but possibly spread over fewer districts. If district variation is more important, then collecting data from more districts will be prioritized, with fewer BHUs per district.

**Verification of Health Department data** We will collect data on (1) timing of deliveries and (2) facility readiness in order to assess accuracy of routinely reported data, a priority for the Health Department.

1. *Reported timing of deliveries*:
  The Health Department, specifically PSPU, have agreed to implement a process whereby SMS messages reporting deliveries will be copied to the research team. A fieldworker will then verify the birth timing. Verification will happen in one of three ways:
  i. The fieldworker may already be at the facility observing the labour/delivery as part of our supplementary data collection (see below).
  ii. During daytime hours, the fieldworker will attend the facility as soon as possible.
  iii. During nighttime hours, the fieldworker will attend the facility the following morning and, if necessary, make a home visit (women often return home very promptly, but patient address and phone number are routinely collected by BHUs.)
  The field team will verify a minimum of 30 birth reports per BHU, verifying each one in sequence from the initial entry into each BHU fieldwork period. Verification will involve recording the time of the birth and a set of questions designed to (1) verify who delivered the child and (2) record key indicators of quality of care and (3) patient satisfaction. We are aware that our field team’s presence may prompt staff to adjust their reporting of births via SMS. For instance, the accuracy of timing may improve due to increased attention to detail and/or reduction in any prior fraudulent reporting of nighttime births. Analysis will therefore compare the observed pattern with historical data for the BHUs and districts. It is unlikely that fieldworker presence would prompt a major change in the provision and uptake of nighttime services within the short period of data collection in each locality. The verification process will therefore allow an assessment of both reporting accuracy and the proportion of births taking place at night. This information will inform interpretation of the routine monitoring data discussed above.
2. *Facility readiness:*
  Structured observations, review of documentation and short staff interviews will be used to collect information on key indicators (identified in conjunction with Health Department stakeholders as useful “sentinel” markers from within the full range of indicators currently reported by PSPU). Indicators will cover aspects of physical infrastructure (e.g. labour room privacy; water supplies), stocks and equipment and functioning (e.g. referral records), and also skill mix and numbers of staff. As above, analysis will involve comparison with the routine data for the same BHUs and districts.

**Supplementation of Health Department data** Two important additional dimensions will be examined: (1) work processes and (2) users’ experiences of care.

1. *Work processes:*
  Informed by the programme theory and systems modelling, recognized standards and assessment tools [39–41] and the PARiHS framework [25], we will develop structured data collection tools to capture key indicators at the district and BHU levels via structured observation and short staff interviews. At the district level, the tool will focus on key aspects of governance and management, including human resource allocation; M&E system functioning; financial reporting; record-keeping; and communications with and support to BHUs. At the BHU level, the work processes tool will include indicators of personnel management (job descriptions; supervision arrangements; nighttime security); referral systems (referral protocols; transport system; efficient communication and cooperation between actors in the referral chains); supply chains (forecasting; functional chain of supply); and monitoring and record-keeping.
  In addition, structured clinical observations of patient–provider interactions will be undertaken for a minimum of 30 antenatal care and 30 labour and delivery interactions (from entry to exit) for each BHU.
2. *User perspectives:*
  Exit interviews will be carried out with at least 30 women in each BHU. As far as possible, these will be the same women whose labour/birth experience has been observed. Women will most often be followed up at home a day or two post-delivery. Interviews will not be longer than half an hour and will ask for respondents’ perspectives on staff availability and contact time; staff–patient communication; privacy; information; choice; respect; dignity; and emotional support. Exit interviews will also take the opportunity to ask brief questions about choice of delivery place and whether the woman would have attended the facility after dark, and if not, why not. Sociodemographic data will also be recorded. Data will be collected by clinically trained fieldworkers working in pairs. They will be trained and assessed on WHO clinical standards, use of assessment tools and objective scoring to foster quality.

### Profiling BHUs

“Thumbnail” sketches of each BHU will be developed. A template will be prepared for this purpose informed by the programme theory and system modelling, and this will be populated via a series of brief semi-structured interviews with personnel, observational activity during the course of site visits, and debriefing with data collectors. A particular objective will be to identify local-level innovations, “work-arounds” and “interpretations” of the initiative that appear to be working well. In addition, local people and providers will be engaged in discussions of how things might work better to elicit “change ideas” for later consideration in WP2. These sketches will be combined with quantitative summary measures for BHUs to produce overall BHU profiles, which will be examined qualitatively to determine whether BHU “types” can be identified (reflecting particular expressions of system functioning).

*Data management and analysis* Quantitative data will be analysed using Stata 17 software [37]. For each domain of interest, assessment criteria will be scored, summed and rated using accepted external standards (e.g. “nonfunctional”/“partially functional”/“fully functional” referral system). Indicators will be reported for each BHU, and distributions of BHU ratings will be reported by district and overall for the province. We will also describe the profile of women using BHU facilities in the different districts and overall. Bivariate statistics and multivariable, multilevel modelling will then be used to explore factors associated with better/worse performance on key indicators at the district, BHU and woman levels.

### WP1—module 3: understanding system processes in detail

Addresses research questions:

*Q3: How does the 24/7 BHU initiative work in the dynamics of different settings? What system processes are supporting or undermining successful scale-up?*

*Q4: How does the 24/7 BHU programme theory relate to system influences on scale-up? What are the key areas of misalignment?*

Building on the insights gained via module 2, this module aims to generate a deeper understanding of the system processes that ensue, and how the scale-up of the 24/7 BHU initiative unfolds, in different settings. An institutional ethnography (IE) will be conducted in 4–5 contrasting BHUs selected using the profiles generated above, and including positive deviants, in three districts, their district headquarters offices and the provincial health directorate in Lahore. Increasingly recognized as a powerful tool in implementation research and quality improvement, IE is a qualitative method that examines everyday processes, relationships and interactions at work to identify broader “ruling relations” within the organization and wider socio-ecological system [42, 43]. Data will be generated using a range of formal and informal observations, guided interviews and group discussions. Data generation will be informed, but not constrained, by our prior system modelling work and the PARiHS framework. We will thereby aim for an effective balance between “bottom-up” inductive data generation that excels at revealing the taken-for-granted and unexpected, and a more structured approach that tests hypotheses and guards against the inadvertent oversight of key system elements or processes.

At the BHU level, a period of 4–6 months in each facility will be used to examine in detail how implementation is unfolding on the ground. The research team will start by observing and recording the various activities that constitute day-to-day work of the midwives and other staff and engaging in informal conversations to develop an “emic” view of BHU “life”. Guided in-depth interviews and focus group discussion will be conducted with midwives and other staff to explore in depth their attitudes towards and understandings and experiences of the initiative, particularly provision of round-the-clock care. These exercises will also include reflection on the “journey so far”, engaging staff in the production of a timeline with identification of “highs” and “lows” as another route to understanding system processes. Women, their husbands and families, and wider community members will also be engaged via interviews, group discussions and informal conversations. Service-user perspectives on how the BHU is functioning and the quality of care offered may diverge from staff reports and recognized standards of good practice. Potential changes in relationships and accountability between local providers and communities will also be of interest.

At the district level, a similar approach to “immersion” in the work of the officers will be taken. We will conduct informal and formal interviews and group discussions, shadow officers in their duties and site visits to BHUs, and examine governance and management processes. Our focus will be on both the “hard” and the “soft” elements of the system, seeking to test hypotheses generated via earlier modules. All local actors will also be engaged in discussions of how to improve the initiative and any “change ideas” fed into WP2. It is inappropriate to fix sample sizes in advance in qualitative research, and we will assess saturation of data as fieldwork progresses. In terms of formal, recorded data, we expect to conduct a minimum of 40 professional interviews, 12 focus group discussions and 30 patient/carer interviews. Informal observations and interviews will be far more numerous.

*Data management and analysis* Data generation and analysis will be concurrent and will be guided by an ongoing dialogue with, and refinement of, the programme theory and system modelling. Qualitative data will be collected using audio recorders and detailed note-taking. A database of the transcribed interviews, focus groups and observation notes will be created in Quirkos. Interpretive accuracy will be assessed by peer debriefing within the research team and other colleagues and respondent validation as an ongoing activity [44]. To make the analysis process manageable, within-case analysis will occur first. We will prepare operational memos for each BHU and district. These will follow a loose structure guided by earlier module findings and will document key aspects of “what is/has been going on” in each site. Next, holistic thematic memos will draw out more interpretive claims about “how the system is/has been functioning” in each BHU/district setting and will include excerpts of empirical material in support of claims (e.g. direct quotations; observation notes). Cross-case analysis will then be initiated through the systematic comparison across memos in a series of team analysis workshops to identify patterns and relationships in the data (e.g. processes that amplify or dampen aspects of the initiative). A combination of data tabulation, diagraming and narrative techniques will be used to organize the data into manageable evidence “nuggets”. We envisage an analytical dialogue between the empirical data and the emergent system model. Hypothesized causal processes identified in the system model via earlier modules will be tested against ethnographic data and confirmed, refined or refuted. Plus, empirical data will be mapped onto the system model, so that, where necessary, new elements and relationships will be added to the model.

### WP2

Addresses research questions:

*Q4: How does the 24/7 BHU programme theory relate to systems influences on scale-up? What are the key areas of misalignment?*

*Q5: How can system elements and processes be manoeuvred to improve implementation of the initiative overall and for subgroups of women who are poorly served?*

*Q6: What key system elements and processes may need to be monitored and addressed to sustain the initiative going forward?*

The goal of WP2 is to identify feasible and acceptable modifications to the 24/7 BHU initiative that can be tested to assess their promise in supporting improved implementation in a wider range of settings across Punjab. Ongoing dialogue with senior Health Directorate and PSPU staff confirms commitment to innovation and a desire to develop capacity in improvement science techniques.

### WP2—module 1: integrating understanding and identifying plausible improvements

An initial one-day project launch workshop will be held in month 2. This event will engage key policy and practice stakeholders, members of the Policy and Programming Research Stakeholder Group (PPRSG), in the project aims and design and aim to engender a sense of ownership of the project. Ongoing one-to-one and small group meetings will subsequently take place regularly during WP1 to share progress with key policy-makers.

In month 24, a second deliberative workshop will bring together members of PPRSG with district health officers of the sampled districts to discuss findings from WP1 modules 1 and 2 and to generate a shared understanding of the emerging system model and areas of uncertainty. Priorities for investigation during module 3 will be identified. This workshop will also introduce the PDSA cycle approach to improvement and invite early discussion on possible “change ideas”. A key objective will be to gain consensus that the PDSA process will generate learning but cannot promise achievement of desired outcomes; stakeholders must accept the possibility that improvement goals cannot be achieved under current constraints, and that new issues may arise [27]. After this workshop, regular meetings and webinars will maintain stakeholder engagement in the project activities.

In month 38, a two-day deliberative workshop will bring together PPRSG and district health officers to discuss the integrated findings from across the whole of WP1. Data will be presented via evidence summaries using a mix of formats (visual, textual, vignettes, etc.) that will be coproduced with key stakeholders prior to the workshop to ensure clarity and relevance. These will summarize patterns and variations in implementation status; gaps between programme theory and operational realities; processes at work in successful facilities (including local innovations and “work-arounds”); and “change ideas” proposed by different actors in the system. A refined system model will also be shared (visually in causal loop diagrams and in narrative form) that integrates these evidence elements and engages stakeholders in the “systems thinking” approach. We will also provide workshop participants with stimulus by presenting a range of improvement approaches that have been tried in other contexts [45]. An informal and participatory environment will encourage open debate and dialogue. As advocated by Reed and Card [27], the workshop process will promote clear understanding and framing of the system challenges. Workshop participants will then work collectively to identify and elaborate potential ways to enhance system enablers and/or circumvent obstacles. The focus will be on achieving standardized function rather than on following a single form of provision (e.g. round-the-clock care could vary in form, with some BHUs having midwives in situ 24 hours and others operating an “on-call” system). This will produce a list of candidate “change ideas”. Finally, workshop participants will be engaged in a discussion to establish prioritization criteria that will be used to select a small number of “change ideas” to be taken forward. Workshop discussions will be carefully documented and an accessible report prepared immediately afterwards.

### WP2—module 2: plan, pilot and document modifications

The next step will involve team members and senior managers in IRMNCHN in (i) identifying a district and 2–3 BHUs within which a PDSA [26] approach is to be undertaken and (ii) selecting priority “change ideas” to be piloted. Action will then move to the district and BHU levels. PDSA cycles will be led by local managers and practitioners at the district and/or BHU level, with hands-on support from the research team. The steps are as follows:

(i) Establish the local team, roles and a shared understanding of the PDSA approach.
(ii) Develop a “proof-of-concept” piloting plan defining the elements of the *24/7 BHU* initiative to be adjusted, articulating the “logic” of the modifications and establishing success criteria.
(iii) Define the scope and duration of the pilot (anticipate quick development cycles, 6–8 weeks).
(iv) Develop a clear work plan for successful implementation of the modification(s).
(v) Identify and minimize any risks to the pilot.
(vi) Determine a suitable monitoring system (likely to be both quantitative and qualitative).
(vii) Implement the strategy.
(viii) Evaluate effects quantitatively and qualitatively against the success criteria.
(ix) Collectively discuss implications and agree on next steps (abandon, modify, expand).
(x) Repeat cycle with new or refined “change idea”.

In addition to the M&E embedded within the PDSA cycles, team members will document the process as it unfolds through observation and informal interviews with the key actors in order to capture key learning. Products from WP2 will be coproduced by the research team and the policy/practice partners and will include the following:

- Documentation of each PDSA cycle undertaken describing the “change idea”, the ensuing system changes and outcomes observed, learning and follow on actions. We will experiment with visual, text-based and short video capture as ways of sharing learning across theprogramme.
- A reflective account of the PDSA process as a whole as it was undertaken and experienced across the sites and recommendations for future use of this approach by the government and PSPU.
- A longer-term improvement strategy capturing “change ideas” generated but not piloted during this project and system aspects warranting further attention as the 24/7 BHU initiative continues.

## Discussion

### Anticipated achievements of the research

The ultimate aim of this study is to improve the chances of women in Punjab having a safe pregnancy and childbirth, free from avoidable morbidity and mortality. The study aims to contribute to improved access to skilled and respectful birth attendance and emergency obstetric care for all women, but particularly for disadvantaged women in remote, rural Pakistan, when they need it. Such improvements in maternal health will also benefit women’s families and their communities.

A major impact of this study will be to enable the Punjab Health Department to deliver high-quality, round-the-clock maternal health services in its first-level BHU facilities in remote, rural areas. The study will coproduce significant new understandings of the system in which the 24/7 BHU initiative is implemented, and actionable knowledge that will highlight ways the implementation process might be modified to enable BHUs to meet service provision goals. By working closely with members of the Health Department's Implementation and Monitoring & Strategy branches, this study will foster recognition of the value of mixed-methods research and the importance of empirical evidence in health system change. The study will also reveal obstacles and issues that require further attention and will aim to foster a culture of openness and “learning from failure” as well as success.

The study will help build research capacity. Junior researchers recruited to work on the project will receive a range of training and mentoring in concept development, methods, developing manuscripts and communicating research findings to varied audiences. In Pakistan, government technical and managerial staff will develop an appreciation of the role and importance of evidence in policy and management decisions.

This study will also have an impact on a wider level, producing insights that will be relevant for other South Asian and LMICs that experience similar challenges of programme scale-up, health equity, gender and health, quality improvement and the delivery of maternal health services to remote and marginalized communities. Our insights will help policy actors in these geographical regions identify and act upon what is required to successfully merge pilot projects into existing, but often fragile, health systems. The study will make an important contribution to the ways in which we can conceptualize scale-up processes. This is a relatively new area, and our use of systems thinking alongside implementation and improvement frameworks is innovative. Methodologically, we anticipate significant new learning across three areas: integration of routine and new qualitative and quantitative data into “soft” systems modelling approaches; PDSA improvement approaches (which to date have been used on only a very limited basis within LMIC contexts and within community health service settings); and coproduction of systems understanding and improvement modifications with policy and practice stakeholders.

### Strategy for uptake

We aim to achieve the following objectives when communicating our research:

- to provide academics, policy and practice stakeholders, and the public with evidence on the degree of success in the scale-up of the 24/7 BHU initiative, variation across the province, why we see the patterns we do, and likely routes to better performance;
- to showcase new conceptual tools and analytical approaches that can enhance research into scale-up of healthcare initiatives inLMICcontexts;
- to provide a model for the conduct ofcoproducedimplementation research in LMIC settings; and
- to develop understanding among policy stakeholders on systems theory and methods for the analysis of complex health systems and improvement initiatives within them.

We identify three main audiences for this project: (1) academics from multiple health and social science disciplines; (2) policy stakeholders from across local, provincial and national governments; national and international third-sector organizations; donor agencies; practitioners and professional bodies; and the private sector; and (3) the lay public whose lives are affected by the quality and availability of maternal healthcare services.

Within our coproduction of knowledge approach, we will empower district-level, frontline staff to draw upon their grounded perspectives to generate actionable knowledge. By engaging these workers, we will empower district-level systems understanding and improvement modifications. An additional impact of this research will be the introduction of the PDSA improvement approaches in LMIC settings, which to date have only been used on a very limited basis.

## Data Availability

The datasets generated and/or analysed during the current study will not be publicly available but are available from the corresponding author on reasonable request.
